# A Coprological Investigation of Endoparasites in One‐Horned Rhinoceros (*Rhinoceros unicornis*) Outside the Chitwan National Park, Nepal

**DOI:** 10.1155/vmi/8885152

**Published:** 2026-07-29

**Authors:** Roshan Babu Adhikari, Madhuri Adhikari Dhakal, Tirth Raj Ghimire

**Affiliations:** ^1^ Department of Zoology, Nepalese Army Institute of Health Sciences (NAIHS), Kathmandu, Bagmati, Nepal, naihs.edu.np; ^2^ Department of Zoology, Alka Health Institute Pvt. Ltd., Lalitpur, Bagmati, Nepal; ^3^ Wildlife Biology, Third Pole Conservancy (TPC), Bhaktapur, Bagmati, Nepal, thirdpoleconservancy.org; ^4^ Department of Microbiology and Research and Development, New Edge Microbials, Albury, New South Wales, Australia; ^5^ Animal Research Laboratory, Faculty of Science, Nepal Academy of Science and Technology (NAST), Lalitpur, Bagmati, Nepal, nast.gov.np; ^6^ Department of Zoology, Tri-Chandra Multiple Campus, Tribhuvan University, Kathmandu, Bagmati, Nepal, tribhuvan-university.edu.np; ^7^ Central Department of Zoology, Tribhuvan University, Kathmandu, Bagmati, Nepal, tribhuvan-university.edu.np

**Keywords:** *Anoplocephala*, *Eimeria*, endangerment, polyparasitism, rhino, stressors

## Abstract

**Introduction:**

The One‐HornedRhino (*Rhinoceros unicornis*) is a vulnerable species that inhabits the lowlands of Nepal. With the existence of several threatening factors, such as poaching, natural disasters, food toxicity, and diseases, their survival and overall health are continuously threatened. In this context, diseases caused by intestinal parasites can serve as a warning sign. However, the occurrence of parasite types and the predicted impacts of parasitism in these megaherbivores remained unclear and questionable in Nepal.

**Aims:**

The current study aimed to determine the presence of endoparasites (protozoa and helminths) inhabiting these hosts, which naturally live freely outside the Chitwan National Park (CNP) in Central Nepal.

**Methodology:**

Sixty fresh fecal samples (*N* = 60) from five different sampling locations along the nearby territories were collected opportunistically using noninvasive methods and transported to the research laboratory. Coproscopy was carried out following standard protocols and laboratory techniques, including direct wet mount, concentration, and acid‐fast staining methods.

**Results:**

The results showed a 100% prevalence rate, with 16 species of intestinal parasites (Protozoa: 5; Helminths: 11). Furthermore, the chi‐square (*χ* ^2^) goodness‐of‐fit test indicated a statistical difference between the frequencies of protozoan (16.7%) and helminth (98.3%) detections (*p* < 0.05, *χ* ^2^ = 5.04, degree of freedom = 1). *Entamoeba* sp. and *Eimeria* spp. were the most frequently recorded protozoa, while *Anoplocephala* spp. and Strongyle were the most frequently recorded helminths. Additionally, monoparasitic infection was not observed, and quadruplet infection (coinfection with four parasite species) represented the most prevalent pattern (25%).

**Conclusions:**

This is the first study to document protozoan and helminth diversity and multiparasitism in free‐ranging rhinos outside protected areas in Nepal. Our findings of 100% prevalence and 16 diverse parasite taxa highlight the need for urgent antiparasitic drug treatment for those rhino populations.

## 1. Introduction

*Rhinoceros unicornis,* Taxonomic Serial Number 625005 (https://www.itis.gov), also known as the greater One‐Horned Rhino (Family: Rhinocerotidae), is an odd‐toed ungulate and mega terrestrial herbivorous mammal. Prehistoric studies suggest that these animals inhabited the flood plains of the Brahmaputra, Ganges, and Sindh rivers, along with their branching tributaries between the Indo–Burmese border in the east up to the Indo–Pakistan border in the west, including the lowland of Terai in Nepal [1]. However, currently, their population is restricted to a few protected areas in Nepal and India [2, 3].

In Nepal, they inhabit the lowlands of the Terai and usually prefer riverine grasslands and forests, floodplains, and adjoining swamps and wetlands [4, 5]. According to a recent National Rhino Count 2021, a total of 752 individual rhinos inhabit around four major protected areas of Nepal, mainly the Chitwan National Park (CNP) (694), followed by Bardia (38), Suklaphanta (17), and Parsa National Park (3) [6]. Although rhinos are the flagship species, their survival and welfare are unlikely to be secure due to multiple threats. Deforestation leading to habitat shrinkage and illegal hunting/poaching are vital anthropogenic factors [7, 8]. Because of several myths spread about rhino horn for its medicinal properties, such as its ability to relieve humans from fever, liver problems, hangovers, cancers, and lack of manliness [9], poachers murder them for their horns.

On the other hand, diseases, predator pressure, territorial conflicts, swamp traps, drowning, and climatic factors exist as natural threats [7, 8]. Thus, rhinos are categorized as a “vulnerable” species under the IUCN Red List and Appendix I of CITES, plus as an “endangered” species under the National Red List of Mammals [3, 10]. In addition to the consequential impact of various threats, intestinal parasitism may be a significant threat. The threats posed by these parasites to rhinos have been documented in a few geographic regions. For instance, gastrointestinal (GI) disorders, including enteritis, impacted guts, and parasitic malnutrition, led to the deaths of rhinos, including their calves, in captivity in many countries, such as India [11] and Tanzania [12]. Unfortunately, Nepal, too, faced a tragic loss of 165 rhinos within the last five years [13], but the leading cause for most of these deaths remains elusive. In these circumstances, examining parasitic diversity and burden can help assess the roles of different intestinal parasites in the status of GI health. Thus, it is a matter of concern for the concerned authorities to deal with the possible effects of parasitism for the efficient act of conservation and management. However, the occurrence and the predicted impacts of the intestinal parasites in these mega herbivores remained unclear and questionable in Nepal.

Although a few authors [14, 15] have listed the helminth fauna in these hosts within protected areas of Nepal, there remains a clear gap in knowledge about protozoan diversity and overall parasitic burden. Additionally, no attempts have been made to determine the prevalence of parasitism among rhinos living outside protected areas. In the CNP, the adjoining buffer zone and community forest alone host more than 10% of the total rhino population [5]. Because rhinos frequently move outside protected areas into adjoining buffer zones, community forests, and even human settlements and agricultural areas, as noted repeatedly, they may acquire a diverse range of parasites and pose zoonotic risks of parasitism in peripheral human settlement areas. In this context, the One Health Approach of understanding parasites and parasitic diseases in wild and domestic animals, nearby humans, and the local environment, including soil, water, and vegetation, is critical. However, we have not understood the prevalence and diversity of parasites in the local rhino population that could be critical for One Health. Therefore, investigating parasites and their loads outside the park would provide information on rhinos’ GI health and the potential zoonotic risks they pose to human health. Hence, the current study is the first exhaustive coprological survey designed to investigate the prevalence, diversity, and abundance of protozoa, helminths, and multiparasitism in these megaherbivores living freely outside the CNP.

## 2. Materials and Methods

### 2.1. Study Area

**FIGURE 1:**
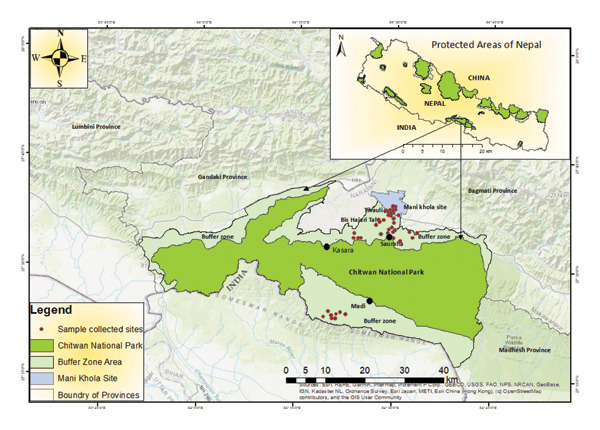
Map of the study area.

**FIGURE 2:**
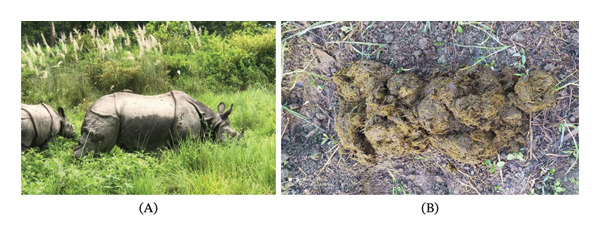
Rhinos grazing outside the Chitwan National Park and dung in the public croplands. (A) Rhinos grazing. (B) Rhino’s feces in the agricultural (garlic) field.

The current study was conducted outside the CNP, a World Heritage Site in the Chitwan district in central Nepal (Figure 1). The area falls under the subtropical to tropical lowlands of inner Terai with an average annual temperature and precipitation ranging from 16°C to 30°C and 154.6 mm, respectively (Retrieved 5^th^ August 2022 from https://geotsy.com/en/nepal/chitwan-national-park-11091/weather-and-climate). It supports a wide range of vegetation, such as tropical sal forest (*Shorea robusta*), riverine forest, khair (*Acacia catechu*), sissoo (*Dalbergia sissoo*), simal (*Bombax ceiba*), and smaller to taller grasses [16]. The study area includes the adjacent buffer zone forests (Tikauli Buffer Zone Community Forest, Milijuli Buffer Zone Community Forest) and community forest areas (Panchakanya Community Forest, Kalika Community Forest, Jaldevi Community Forest, Kumroj Community Forest, Baghmara Community Forest, and Chitrasen Community Forest), especially in the northeastern part of CNP. These areas serve as natural extensions for the park and are preferable habitats for rhinos. The core areas include the nearby territories of Tikauli Taal, Twenty Thousand Lake, Rhino Lake, the river basins of Khageri, Panchanadi, Monikhola, Budi Rapti, and Rapti River along their branching tributaries. Since these areas serve as ecotone regions, humans, including domestic animals and wild animals, particularly such as rhinos, elephants, and deer, are bound to share the overlapping niches [17]. That is why rhinos in these areas usually invade human settlements, agricultural fields, and pasture lands and defecate over there (Figure 2).

### 2.2. Fecal Sample Collection, Preservation, and Transportation

Rhinos exhibit a peculiar behavior of sharing common latrines handed down by their ancestor [18]; hence, large feces can easily be noticed in their preferred defecating sites. The preferable sites are usually the nearby territorial boundaries, water bodies, feeding areas, and their usual paths [19].

From November 2020 to March 2021, we followed these preferable sites within our five major sampling locations (Sauraha, Tikauli, Monikhola, Bharatpur, and Madi) and opportunistically collected 60 fecal samples noninvasively. Since rhinos are more active early morning and late afternoon, each sampling sites and the nearby territories of prone rhino areas were visited on daily basis from (7–9 am, Nepalese time) in the morning and evening (3:30–5:30 pm, Nepalese time) in assistance with the local villagers, or Community Forest User Groups (CFUGs), and eco‐guards to locate small to large crash of rhinos. Only the fresh fecal samples with a moist, warm, shiny surface were considered for investigation. The samples were first photographed and examined macroscopically to ensure the presence or absence of blood, mucus, and segments of worms in them. The upper layer of the feces was removed, and the portion above the ground was collected using a gloved hand and a spatula into 50‐mL sterile vials. For fecal preservation, 2.5% weight/volume (w/v) potassium dichromate solution (K_2_Cr_2_O_7_) was added to the vials immediately at the sampling sites. All the vials are then transported to the Animal Research Laboratory of Nepal Academy of Science and Technology, Lalitpur, Nepal, for microscopic examination and further investigation.

### 2.3. Laboratory Processing and Examination

We employed four different techniques for the examination of fecal samples. About 10 g of the fecal sample was taken in a beaker, and 15 mL of normal saline (0.9% w/v NaCl) was added to it and stirred vigorously. The mixture was then filtered through a metal tea strainer into a Petri dish, and the filtrate was used for microscopic examination.

#### 2.3.1. Direct Wet Mount Technique

The filtrate was carefully stirred, and a single drop was placed on the glass slide with or without Gram’s iodine stain. The smear was then covered with a coverslip and observed under 10x and 40x magnification of the objective lens [20]. Additionally, to improve clarity and ensure that no details were overlooked, the entire slide was thoroughly inspected using a systematic zigzag (left‐to‐right, top‐to‐bottom) pattern [21].

#### 2.3.2. Saturated Salt Flotation Technique

This technique was employed to float primarily coccidian oocysts (e.g., *Eimeria* spp.), *Anoplocephala* eggs, and nematode eggs, which readily float in the flotation medium. About 2 g of the filtrate was thoroughly mixed in 12 mL of normal saline in a beaker. It was then poured into a conical centrifuge tube and centrifuged at 1200 revolutions per minute (rpm) × 5 min [22]. After discarding the supernatant, the tube was filled with flotation medium (45% w/v NaCl; specific gravity ≈ 1.20) and recentrifuged (1200 rpm × 5 min). Then, without discarding the supernatant, the flotation medium was added dropwise to fill the tube [22]. Finally, a coverslip was placed at the opening of the tube, touching the flotation medium. After 10 min, the coverslip was carefully removed and placed on a glass slide for microscopic examination at 10x and 40x magnification of the objective lens with or without Gram’s iodine [23, 24].

#### 2.3.3. Formalin–Ethyl Acetate (FEA) Sedimentation Technique

This method was employed primarily for detecting protozoan cysts, oocysts of *Cryptosporidium* sp., and trematode eggs that do not float in the flotation medium. For this method, the sediment obtained after a single centrifugation step was collected, and 10 mL of 10% formalin and 4 mL of ethyl acetate were added [25]. The sample was then recentrifuged (1200 rpm × 5 min), and the supernatant was discarded. A single drop of the final sediment was then examined at 10x and 40x magnification of the objective lens of the microscope using Gram’s iodine.

#### 2.3.4. Acid‐Fast Staining (AFS) Technique

This technique was employed only for samples that were microscopically positive for *Cryptosporidium*‐like oocysts, either on direct smear or by FEA sedimentation. For AFS, the sediment obtained after FEA sedimentation was used to prepare thin smears [26]. The smear was initially dried at room temperature and fixed in absolute methanol for 2 min. It was then stained with carbol fuchsin for 15 min and gently rinsed with distilled water and acid alcohol [27]. The smear was again restained with malachite green for a minute and then washed with distilled water. Finally, it was dried at room temperature and examined at 100x magnification of the objective lens with immersion oil.

### 2.4. Estimation of Parasitic Burden/Severity of Infection

In order to estimate the burden of parasitic infection, we employed an “A 2‐Cell McMaster Counting Slide” (Hawksley and Sons Ltd). It was measured by quantifying the number of oocysts of protozoa and eggs of helminth parasites released per gram of feces. The procedure is based on the instructions provided by the manufacturer and the literature previously explained [28, 29].

### 2.5. Parasite Identification

Microscopic images of all the parasitic stages, such as cysts, oocysts, and trophozoites of protozoa, as well as eggs and larvae of helminths, were captured with a camera (SXView 2.2.0.172 Beta (Nov 6, 2014) Copyright (C) 2013‐2014), accompanied with the microscope (Optika Microscopes Italy, B‐383PLi). Morphometric analysis was performed using ImageJ 1.51 k (National Institute of Health, USA) software, and the parasitic identification was based on the literature previously described [30, 31]. Additionally, *Fasciola* spp. and *Paramphistomum* spp. were identified based on the staining property with methylene blue [32]. *Fasciola* spp. retain a golden yellow, while *Paramphistomum* spp*.* remains colorless with a methylene blue stain [33].

### 2.6. Data Analysis

All the parasitological data generated in the current study were encrypted and entered into Microsoft Excel 2016 spreadsheets. The percentage prevalence of individual parasites was calculated by dividing the total positive cases of individual parasites by the total sampling population and then multiplying the result by 100.

(1) Prevalence%=total positive casestotal sampling population×100.

The diversity of intestinal parasites was calculated by counting the numbers of parasites present in the total samples. Similarly, the data were analyzed using GraphPad Prism 2007 and specific test; a Pearson’s chi‐squared (*χ* ^2^) test for goodness of fit was used to compare category counts of parasite species within protozoa (*Balantidium coli*, *Cryptosporidium* sp., *Giardia* sp., *Eimeria* spp., and *Entamoeba* sp.) or helminths (*Anoplocephala* spp., Ascarid, *Fasciola* spp*., Habronema* sp., hookworm, Oxyurid*, Paramphistomum* spp*., Schistosoma* sp*.,* Strongyle*, Strongyloides* sp*.,* and *Trichuris* sp.), and protozoa and helminths. In this context, the tests were considered significant for probability values less than 0.05 at different degrees of freedom (DF) (*p* < 0.05) and 95% confidence interval (5% significance level).

## 3. Results

**FIGURE 3:**
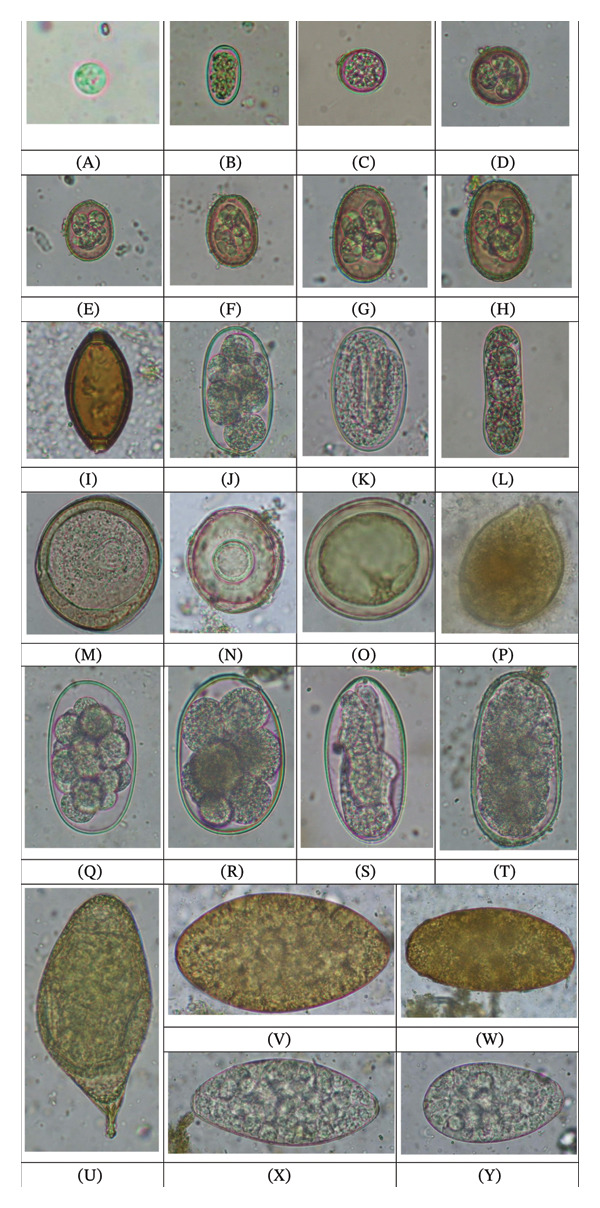
Gastrointestinal parasites identified in One‐Horned Rhinos (microscopic photographs under 40x magnification of objective lens of a microscope). (A) Cyst of *Entamoeba* sp. (8 × 8 µm) after FEA sedimentation technique. (B) Oocysts of *Eimeria* sp. 1. (22 × 12 µm) after direct wet mount technique. (C) Oocyst of *Eimeria* sp. 2. (20 × 18 µm) after direct wet mount technique. (D) Oocyst of *Eimeria* sp. 3. (26 × 26 µm) after flotation technique. (E) Oocyst of *Eimeria* sp. 4. (31 × 24 µm) after flotation technique. (F) Oocyst of *Eimeria* sp. 5. (36 × 25 µm) after flotation technique. (G) Oocyst of *Eimeria* sp. 6. (41 × 26 µm) after flotation technique. (H) Oocyst of *Eimeria* sp. 7. (40 × 26 µm) after flotation technique. (I) Egg of *Trichuris* sp. (61 × 33 µm) after FEA sedimentation. (J) Egg of hookworm (61 × 39 µm) after FEA sedimentation technique. (K) Egg of *Strongyloides* sp. (56 × 35 µm) after flotation technique. (L) Egg of *Habronema* sp. (45 × 17 µm) after flotation technique. (M) Egg of *Anoplocephala* sp. 1 (91 × 84 µm) after flotation technique. (N) Egg of *Anoplocephala* sp. 2 (53 × 48 µm) FEA sedimentation. (O) Egg of Ascarid (71 × 68 µm) after flotation technique. (P) Trophozoite of *Balantidium coli* (91 × 69 µm) after direct wet mount technique. (Q) Egg of Strongyle 1 (75 × 43 µm) after flotation technique. (R) Egg of Strongyle 2 (80 × 63 µm) after flotation technique. (S) Egg of Strongyle 3 (85 × 41) after direct wet mount technique. (T) Egg of Strongyle 4 (92 × 47 µm), 400x after FEA sedimentation technique. (U) Egg of *Schistosoma* sp. (143 × 68 µm) after FEA sedimentation technique. (V) Egg of *Fasciola* sp. 1 (178 × 94 µm), 400x after FEA sedimentation technique. (W) Egg of *Fasciola* sp. 2. (157 × 78 µm) after FEA sedimentation technique. (X) Egg of *Paramphistomum* sp. 1 (144 × 67 µm) after FEA sedimentation technique. (Y) Egg of *Paramphistomum* sp. 2. (115 × 65 µm) after FEA sedimentation technique.

We recorded that 100% (60/60) of the fecal samples examined were positive for the presence of parasites. Altogether, 16 diverse species of parasites were reported that included both single‐celled protozoa, such as *B. coli*, *Cryptosporidium* sp., *Giardia* sp., *Eimeria* spp., and *Entamoeba* sp., and helminths, including *Anoplocephala* spp., Ascarid, *Fasciola* spp*., Habronema* sp., hookworm, Oxyurid*, Paramphistomum* spp*., Schistosoma* sp*.,* Strongyle*, Strongyloides* sp*.,* and *Trichuris* sp. Among these parasites, *Entamoeba* sp. was the most frequent among protozoa, while *Anoplocephala* spp. and Strongyle were the most frequently recorded helminths. The chi‐square goodness‐of‐fit test indicated a statistical difference between the frequencies of protozoan (16.7%) and helminth (98.3%) detections (*p* < 0.05, *χ* ^2^ = 5.04, DF = 1). In addition, the prevalence of different protozoan species (*p* < 0.0001, *χ* ^2^ = 35.00, DF = 4) indicated a strong heterogeneity in protozoan detections among taxa. Similarly, the prevalence of different helminth species (*p* < 0.0001 (*χ* ^2^ = 96.3, DF = 10) denoted a significant heterogeneity in detections among taxa. It is worth noting that 9 of 16 reported parasites have zoonotic potential. This included *B. coli*, *Cryptosporidium* sp., *Giardia* sp., *Entamoeba* sp., *Fasciola* spp., hookworm, *Schistosoma* sp*., Strongyloides* sp*.,* and *Trichuris* sp. (Table 1) (Figure 3).

**TABLE 1:** Gastrointestinal parasites and their prevalence (%) in the fecal samples of One‐Horned Rhinos outside the Chitwan National Park (*N* = 60).

Classes of GI parasites	Parasites detected	Prevalence (%)	Statistical values
Sarcodina	*Entamoeba* sp.	27 (45)	*p* < 0.0001 (*χ* ^2^ = 35.00, DF = 4)
Mastigophora	*Giardia* sp.	1 (1.7)	
Ciliata	*Balantidium coli*	4 (6.7)	
Coccidia	*Cryptosporidium* sp.	20 (33.3)	
	*Eimeria* spp.	18 (30)	

Trematoda	*Fasciola* spp.	10 (16.7)	*p* < 0.0001 (*χ* ^2^ = 96.3, DF = 10)
	*Paramphistomum* spp.	47 (78.3)	
	*Schistosoma* sp.	10 (16.7)	
Cestoda	*Anoplocephala* spp.	58 (96.7)	
Nematoda	Ascarid	5 (8.3)	
	Hookworm	25 (41.7)	
	Strongyle	50 (83.3)	
	*Strongyloides* sp*.*	9 (15)	
	*Trichuris* sp.	2 (3.3)	
	Oxyurid	13 (21.7)	
	*Habronema* sp.	9 (15)	

Total protozoa		37 (16.7)	*p* < 0.05 (*χ* ^2^ = 5.04 , DF = 1)
Total helminths		59 (98.3)	

*Note: p*: probability, *χ* ^2^ = chi‐square for goodness of fit.

Abbreviation: DF, degree of freedom.

Considering the pattern of parasitic infection, monoparasitic infection was not observed and all the fecal samples were reported with more than one species of parasites. A mixed pattern of parasitic infection with four different species of parasites (quadruplet infection) was the highest (25%), followed by hexuplet (20%) and pentuplet infection (16.7%), while double infection (1.7%) was the least.

Additionally, we also recorded seven morphotypes of *Eimeria* spp. (size: 20–41 × 12–26 µm), two morphotypes of *Anoplocephala* spp. (size: 53–91 × 48–84 µm), two morphotypes of *Fasciola* spp. (size: 157–178 × 78–94 µm), two morphotypes of *Paramphistomum* spp. (size: 115–144 × 65–67 µm), and four morphotypes of Strongyle eggs (size: 75–92 × 41–63 µm), suggesting the demonstration of a greater variety of parasites (Figure 3).

The estimation of parasitic burden as measured by the number of eggs/oocysts in each gram of feces (EPG/OPG) using the McMaster technique revealed that oocyst count per gram of feces of *Eimeria* spp. ranged from 100 to 1500. Similarly, the burden of Strongylid nematode was the highest (200–2400), and that of the *Trichuris* sp. was the least (100–200) (Supporting Table 1).

## 4. Discussion

This is the first study to document protozoan and helminth diversity and multiparasitism in free‐ranging rhinos outside protected areas in Nepal. The current finding of 100% (60/60) prevalence of intestinal parasites is on par with findings from India (100%; *n* = 16) [34], but in the captive population. However, it is higher than previously reported in wild populations from Nepal (CNP and BNP) (82.5%–91%) (40–100) [14, 15] and India (61.9%; *n* = 84) [35]. The dissimilarity in prevalence rates might be attributed to differences in sample size, sampling geography, sampling seasons, and the specific laboratory techniques used for detecting parasitic bodies in fecal smears.

Compared with the previous studies [14, 15, 36] conducted in CNP in Nepal, the prevalence and diversity of intestinal parasites are higher in the current rhino population. Even though it is tough to explain the different parasite rates, a few assumptions can be made. First, there is a difference in habitat and grazing areas. Since livestock grazing is practiced in various parts of the community forest and buffer zone forests [32, 37], rhinos are bound to share their pastures and water bodies with these animals. Close interactions with not only domestic animals but also other wild animals, especially while grazing, enhance the frequency of exposure of rhinos to intestinal parasites [38]. Notably, open defecation practices in some parts of the forest may be a crucial factor, as evidenced by the detection of a high prevalence of human parasites, including *Entamoeba* sp., *Cryptosporidium* sp., *Giardia* sp., and hookworm, in the current rhino population. However, further molecular studies are needed to verify this notion.

The second assumption is that there is greater anthropogenic pressure/stress on rhinos outside the CNP. Higher or repeated exposure of an animal to stressors will permanently mark an elevated level of their stress hormone (glucocorticoid) [39]. These result in a continual decline in the immune efficacy of the host and enhance their susceptibility to greater intensity of parasitic infection [39, 40]. In these circumstances, evidence of human–rhino conflict in the study areas [41, 42] may be a critical stressor, resulting in the acquisition of diverse parasites. The primary drivers of stress in rhinos are habitat shrinkage, livestock pressure, poaching, chasing, pitfall traps, retaliatory poisoning, and electrocution [8, 43]. Additionally, crop raiding on agricultural fields contaminated with excessive levels of pesticides, insecticides, and inorganic fertilizers, and the consumption of water from sources drained by agricultural runoff might also be critical for the GI health of the rhinos; however, further study is needed to verify this notion.

The current study also found a significantly higher prevalence of helminths over protozoa. Among the helminths, *Anoplocephala* spp., well known to cause mucosal inflammation, edema, and ulceration in the herbivore hosts [44], represented the only cestode and the most frequently detected parasite in this investigation. The current prevalence rate (96.7%) was comparatively higher than previous findings within CNP Nepal (16%) [14] and in India (56%) in captivity [34]. However, such a huge prevalence warrants caution since this tapeworm is speculated to be the cause of the death of 18 rhinos, aged between 3 and 5 years, over the past two years in Jaldapara National Park, in India [45]. Studies suggest that four different species of the tapeworm, such as *Anoplocephala vulgaris, A. diminuta, A. latissima,* and *A. gigantea,* are known to occur in rhinos [46–49], suggesting a wider diversity and the predominance of Anoplocephalidae tapeworms in rhinos globally. Since oribatid mites are the intermediate hosts of these tapeworms [50], infection occurs when rhinos inadvertently consume these mites containing infective cysticercoids while grazing on the pasture.

Additionally, we reported the presence of Strongyle eggs in 83.3% of the samples. This prevalence rate was higher than previously reported from Nepal (15%–60.61%) [14, 15]. However, as in our result, their captive population in India was highly infected with this nematode (94%) [34]. Furthermore, an adult in the zoo in Sri Lanka was also found heavily infected (EPG: 2300) with this nematode [51]. Since rhinos are herbivorous, grazing on contaminated pastures and agricultural fields might have contributed to their reproductive success. Interestingly, several Strongylid nematodes, including *Khalilia, Kiluluma, Murshidia,* and *Quilonia,* have been reported from white rhinoceros (*Ceratotherium simum*) and black rhinoceros (*Diceros bicornis*) previously [47]. This is why our findings of several morphotypes of these nematodes suggest the need for further molecular studies to identify the species. We also reported hookworm infection in about 41.7% of the fecal samples. Formerly, human strains of hookworm (*Necator americanus*) have been reported in adult black rhinoceros (*D. bicornis*) [52] and in wild‐caught Indian rhinoceros (*R. unicornis*) [53].

On the other hand, the current prevalence report of nematodes such as Ascarid (8.3%) and *Strongyloides* sp. (15%) was approximately 2–4 times lower than previously reported in Nepal. Notably, *Trichuris* sp., Oxyurid sp., and *Habronema* sp. were our unique findings. The occurrence of nematodes such as *Trichuris* and Oxyurid may be a common phenomenon. However, the presence of *Habronema* sp., a well‐known vector‐borne parasite of horses, in 15% of the fecal samples is concerning, as it causes habronemiasis, a condition characterized by tumors in the stomach and granular dermatitis (summer sores) in horses [54]. Even though muscid flies (houseflies and stable flies) act as intermediate hosts for these parasites for their development in horses, for rhinos, the host might be *Rhinomusca dutoiti,* which develops entirely in the dung of rhinos [48]. Considering the digenetic trematodes, the prevalence report of *Paramphistomum* spp. (78.3%) was the highest. These rumen flukes are responsible for causing acute paramphistomosis in herbivores that include clinical symptoms such as diarrhea, anorexia, dehydration, cachexia, apathy, perceptual alternation, and even death, especially in young animals [55, 56]. Their current findings were comparatively higher than the previous report from Nepal (30.30%–31%) [14, 15] and India (46%) [35] but were lower than the findings from their captive population in India (100%) [34] and Indonesia (90%) [57]. Furthermore, one in two rhinos at a zoo in Bangladesh was also infected with this fluke [58], indicating that rhinos are the potential hosts for this trematode. Since freshwater snails of the genus *Planorbis* and *Lymnaea* act as intermediate hosts for the completion of their life cycle [55, 59], the seasonal floods in the existing rivers and rivulets each year during monsoon provide a suitable environment for their development and existence in the study area.

Other flukes, such as *Fasciola* and *Schistosoma,* were also reported in the current study. Noting the same prevalence rate (16.7%), the presence of *Fasciola* spp. in our study was slightly higher than previously reported within CNP (14%) [14]. However, it was lower than that reported in wild Javan rhinos in Indonesia (44%) [60]. Fasciolids (*Fasciola hepatica, F. gigantica, Fasciolopsis buski, Fascioloides magna,* and *F. jacksoni*) usually parasitize large herbivorous mammals, including rhinos, and are associated with considerable morbidity and mortality in the megaherbivores [11, 60]. Hence, the presence of *Fasciola* spp. is a matter of concern. Since the current rhino population is free‐ranging, grazing in marshy areas, and frequently exposed to associated risk factors, such as metacercaria larvae and aquatic host plants, this exposure may have contributed to the significant acquisition.

It is worth noting that our *Schistosoma* sp. (16.7%) findings were lower than previously reported from CNP (50%) [61]. However, it was slightly higher than reported in Nepal [15] and Indonesia [60] in wild Javan rhinos, where the prevalence rate was almost 12%. Unfortunately, previous studies [14, 36] from Nepal did not document the presence of this fluke in the rhinos within the CNP. Since the infective cercariae remain free‐swimming in the water bodies [62], rhinos acquire infection through swimming, wallowing, and even drinking contaminated water. These flukes are well known to cause anemia, retardation of growth, and digestive and reproductive impairment in domestic hosts [63]. In this scenario, it would be interesting to determine how parasitism would favor large wild herbivores, suggesting a need for further histopathologic studies in the heavily infected population.

Regarding protozoa, we first reported the presence of *Entamoeba* sp. in fecal samples from rhinos in Nepal. However, the presence of amoeba had already been documented in the Javan rhino in Indonesia [31]. A higher prevalence of *Entamoeba* sp. (45%) in rhinos may be critical, as *Entamoeba* is presumed to be pathogenic in these hosts [64]. Among various amoeba strains, *E. histolytica* primarily parasitizes human hosts and is the second most common cause of death in humans with zoonotic potentialities [65]. Nevertheless, the presence of *E. bovis* has been genetically documented in wild herbivores, such as wild deer [66] in Australia and wild water buffaloes in the same region [67], indicating an urgent need for molecular studies of amoebas in these hosts in Nepal.

Similarly, two different coccidia, *Cryptosporidium* sp. (33%) and *Eimeria* spp. (30%), were also reported in the current study. The finding of *Cryptosporidium* oocysts in One‐Horned Rhinos is unique in Nepal; however, these oocysts were previously recorded in the feces of Javan rhinos in Indonesia [31]. Furthermore, the current findings of *Eimeria* spp. (30%) were slightly higher than reported in Nepal previously [14]. Both of these coccidia are reported to have a profound pathogenic effect and even cause mortality in many younger and immunocompromised aging domestic hosts [68–70]; their presence in similar age groups of rhinos could be problematic.

*B. coli* (6.7%) is the only ciliate reported in the present study. These ciliates had previously been reported in rhinos from various geographies, including Bangladesh [58], Sri Lanka [51], Indonesia [31], and India [53], suggesting that *B. coli* is a common parasite of these hosts.

Notably, all the samples examined were positive for multiple parasite species. No single infection was noted. In general, multiparasitism in the wild is a rule rather than an exception and is an integral part of a broad model of parasite assemblages [71]. Coinfection usually favors those hosts inhabiting large geographical areas with greater population density [71]. Multiparasitism with protozoan–protozoan or helminth–helminth species is known to exacerbate immunopathology in naturally infected hosts [72, 73], in experimental animals [74], and in humans [75], thereby increasing the severity of infection. However, negative interactions among the coinfecting parasites can also occur [76]. In our case, the maximum number of rhinos coinfected with multiple protozoa and helminths at a time has moderate to heavy parasitic burdens, as estimated by OPG/EPG, suggesting greater virulence and disease severity in these hosts. However, it remains unclear how the interactions among protozoa–protozoa, helminth–helminth, or protozoa–helminth parasitic communities within an individual mega herbivore, such as a rhino, affect disease outcome. Thus, a detailed histopathologic examination is warranted to further verify this notion. Nevertheless, it must not be ignored that coinfection with intestinal parasites and other pathogens, such as bacteria or viruses, can act synergistically in a host and result in deleterious health conditions [77–79].

We truly acknowledge that the current coprological survey is subject to a few limitations. First, a single fecal sample per animal was used to assess the intestinal parasites. As excretion of parasites via feces can vary with respect to time, duration of infection, and parasite, reliance of single sample may result in the underestimation or overestimation of true prevalence and diversity. Second, the use of opportunistic fecal sampling in the wild may lead to the exclusion of heavily infected or potentially healthy animals or vice versa. This could introduce sampling bias and limit the accuracy of the findings, thereby offering only a partial representation of parasite distribution. Third, the sole reliance on morphometric analysis, owing to the absence of a standard molecular laboratory, limited identification to the genus level. Such identifications may not fully reflect the actual parasite diversity, as it limits our ability to distinguish among morphotypes of parasites such as *Eimeria* spp., *Fasciola* spp., *Paramphistomum* spp., *Anoplocephala* spp., and the Strongylid group, as detected via microscopy. However, we have strictly ensured quality control throughout our fecal sampling and copromicroscopic examination, and our findings offer valuable insights that could inform wildlife conservation strategies and guide integrated animal health programs both outside and within the national parks.

## 5. Conclusions

In conclusion, rhinos outside the CNP harbor a higher prevalence and greater diversity of intestinal parasites than reported in any previous studies in Nepal. Additionally, parasites such as *Entamoeba* sp., *Cryptosporidium* sp., *Giardia* sp., *B. coli*, *Trichuris* sp., and *Habronema* sp. have been reported for the very first time in the Nepalese Rhino. Our findings suggested that the presence of anthropogenic and natural stressors, and the sharing of overlapping niches with domestic animals and humans, remained the critical risk factors for parasitosis. Even though the number of rhinos in Nepal has recently peaked, strategic planning and conservation efforts are concentrated only within protected areas, and no emphasis has been placed on parasitic threats. Therefore, to manage rhinos sustainably, government bodies, including park veterinarians, CFUGs, local people, and wildlife conservationists, should follow integrated conservation strategies not only within protected areas but also beyond them. Although the current cross‐sectional study determined strong heterogeneity and statistical significance among protozoan or helminth species or between protozoa and helminths in small samples, future studies incorporating molecular techniques on large fecal and environmental samples are recommended to achieve more accurate species‐level identification with various statistical tools and techniques, accompanied by mathematical modeling, and improve the resolution of parasite characterization. This would be more practicable for the conduction of future research on One Health strategies so that the epidemiology of parasites and parasitic diseases in the habitat of One‐Horned Rhinos will be better understood. The strategies should include research and prevention programs, including regular examinations of environmental, human, and rhino health, and supplementation with antiparasitic drugs after careful assessment through controlled trials to manage the impact of diverse protozoa, helminths, and multiparasitism in these hosts.

## Author Contributions

R.B.A. conceptualized the project, worked in the field and laboratory, developed the methodology and data analysis, prepared the original draft, and reviewed and edited the manuscript.

M.A.D. conceptualized the project, analyzed data, wrote, reviewed, and edited the manuscript.

T.R.G. conceptualized the project, provided laboratory resources, analyzed data, reviewed, and edited the manuscript.

## Funding

No funding was received for this manuscript.

## Disclosure

All authors approved the final version of the manuscript.

## Ethics Statement

The authors declare that the study was conducted on naturally infected fecal samples of One‐Horned Rhinos. No experimental infection was established during this research work. None of the animals was touched or harmed during the study. The required permission for collecting the fecal samples was obtained from the Government of Nepal, Department of Forest and Soil Conservation (Permission No. 159/077/078) and Division Forest Office, Bharatpur, Chitwan (Permission No. 852/077/078).

## Conflicts of Interest

The authors declare no conflicts of interest.

## Supporting Information

Additional supporting information can be found online in the Supporting Information section.

## Supporting information


**Supporting Information** Supporting Table 1: OPG/EPG in the fecal samples of One‐Horned Rhinos.

## Data Availability

Data are provided within the manuscript or supporting information files.
