# Supplementary material for: A Coprological Investigation of Endoparasites in One‐Horned Rhinoceros (Rhinoceros unicornis) Outside the Chitwan National Park, Nepal
Source: Vet Med Int. 2026 Jul 29;2026:8885152. doi: 10.1155/vmi/8885152 (PMC13417725; doi:10.1155/vmi/8885152)
Supplement: Supplementary file 1 — Supporting Information Supporting Table 1: OPG/EPG in the fecal samples of One‐Horned Rhinos. [file VMI-2026-8885152-s001.docx]

**Supporting Information**

**Supporting Table 1:** OPG/EPG in the fecal samples of one-horned rhinos

| **Parasites** | **Average range of OPG/EPG** |
| --- | --- |
| *Eimeria* spp. | 100–1500 |
| Ascarid | 100–800 |
| Strongyle | 200–2400 |
| *Trichuris* sp. | 100–200 |
| *Strongyloides* sp. | 100–700 |
| Hookworm | 100–1700 |
| *Habronema* sp. | 100–500 |
| Oxyurid | 100–300 |
